# Effect of CYP2C19 Pharmacogenetic Testing on Predicting Citalopram and Escitalopram Tolerability and Efficacy: A Retrospective, Longitudinal Cohort Study

**DOI:** 10.3390/biomedicines11123245

**Published:** 2023-12-07

**Authors:** Mahmood Mahajna, Rami Abu Fanne, Matitiahu Berkovitch, Elias Tannous, Shlomo Vinker, Ilan Green, Ilan Matok

**Affiliations:** 1 Department of Clinical Pharmacy, The Hebrew University, Jerusalem 9112002, Israel; 2 Hillel Yaffe Medical Center, Hadera 3810000, Israel; eliast@hymc.gov.il; 3 Department of Cardiology, Hillel Yaffe Medical Center, Hadera 3200003, Israel; 4 Leumit Health Care Services, Tel Aviv 6812509, Israel; svinker@leumit.co.il (S.V.); igreen@leumit.co.il (I.G.); 5 Department of Clinical Pharmacology and Toxicology, Shamir Medical Center Affiliated with Sackler Faculty of Medicine, Tel Aviv University, Tel Aviv 6997801, Israel; 6 Department of Medical Sciences, Faculty of Medicine, Ben-Gurion University of the Negev, Beersheva 8410501, Israel

**Keywords:** SSRI, pharmacogenetic, adverse effects, phenotype, efficacy

## Abstract

Background—Various antidepressant agents are metabolized by the CYP2C19 enzyme, including Citalopram and Escitalopram. Variation in CYP2C19 expression might give rise to different plasma concentrations of the active metabolites, potentially affecting both drugs’ efficacy and tolerability. Aim—The aim of this study was to evaluate differences in the Escitalopram and Citalopram efficacy and tolerability between different CYP2C19 genotype-based metabolizing categories in outpatients suffering from major depressive disorder (MDD). Methods—In a retrospective, longitudinal cohort study of electronic medical-record data, 283 patients with MDD who were prescribed Escitalopram or Citalopram with the available CYP2C19-genotyping test were enrolled. The primary efficacy end point was adverse drug reactions recorded in the medical files. A proportional-odds, multilevel-regression model for longitudinal ordinal data was used to estimate the relation between the CYP2C19 genotype and adverse drug reactions, adjusting for potential confounding variables and other explanatory variables. Latent-class analysis (LCA) was utilized to detect the presence of clinically significant subgroups and their relation to an individual’s metabolizing status for CYP2D6/CYP2C19. Results—With poor CYP2C19 metabolizers as a reference, for each unit difference in the activity score of the CYP2C19 phenotype, the odds ratio for drug intolerability was lowered by 0.73 (95% credible intervals: 0.56–0.89), adjusting for significant covariates. In addition, applying LCA, we identified two qualitatively different subgroups: the first group (61.85%) exhibited multiple side effects, low compliance, and frequent treatment changes, whereas the second group (38.15%) demonstrated fewer side effects, good adherence, and fewer treatment changes. The CYP2C19 phenotype was substantially associated with the group membership. Conclusions—We found a positive association between the CYP2C19 activity scores, as inferred from the genotype, and both the efficacy of and tolerability to both Es/Citalopram. LCA enabled valuable insights into the underlying structure of the population; the CYP2C19 phenotype has a predictive value that discriminates between low-adherence, low-drug-tolerance, and low-response patients and high-adherence, high-drug-tolerance, and high-response patients. Personalized medicine based on CYP2C19 genotyping could evolve as a promising new avenue towards mitigating Escitalopram and Citalopram therapy and the associated side effects and enhancing treatment success.

## 1. Introduction

Major depressive disorder (MDD) is a common mental disorder that involves persistent feelings of sadness and loss of interest and is associated with an increased risk of suicide, disability in life functioning, and a wide range of chronic physical comorbidities [1].

Selective serotonin reuptake inhibitors (SSRIs), such as Citalopram and Escitalopram, are commonly used to treat patients with depressive disorders and, in recent years, they have been increasingly prescribed [2]. Nonetheless, 30–40% of depressed patients treated with SSRIs exhibit partial clinical remission or experience significant drug-related adverse events that result in low drug adherence, treatment discontinuation, and continuous suffering [3,4].

Every medication can cause side effects, and anti-depressant medications are no exception. There are several classes of antidepressants with near equal clinical efficacies in the treatment of depression [5]. The prescription patterns of antidepressant medications are a function of patient-specific (e.g., preference, past side effects, drug interactions, comorbidities, and age) and drug-specific (e.g., side effects, safety profile, and type of formulation) factors. The most widely prescribed antidepressants are SSRIs, mainly because of their superior tolerability and safety profiles. According to the National Institute for Clinical Excellence (NICE) and Clinical Pharmacogenetics Implementation Consortium (CPIC) depression guidelines, SSRIs are considered the first-line treatment for MDD [6].

SSRIs are subjected to extensive metabolism, with a key role played by cytochrome P450 isoenzymes. Notably, cytochrome P450 is a genetically polymorphic drug-metabolizing enzyme with large interindividual variability. CYP2C19 is a member of the cytochrome P450 enzyme family, with high genetic polymorphism that influences the range of CYP2C19 enzyme activity, with controversial results. The interindividual variability in CYP2C19-mediated metabolism may affect drug concentrations/drug elimination, which might potentially impact both drug efficacy and safety [7]. Pharmacogenetic studies of SSRIs have mainly focused on the impact of the pharmacokinetic aspects on both drug tolerability and safety [8]. In the case of Citalopram and Escitalopram, they are extensively metabolized by the liver, predominantly by the CYP2C19 system, with the derived metabolites considered less potent than the parent compound [9]. On the basis of the CYP2C19 activity range, individuals can be classified into one of five CYP2C19 phenotype categories: (1) extensive metabolizers (EMs) carrying normal-function alleles (CYP2C19*1/*1, *2/*17, *4/*17); (2) intermediate metabolizers (IMs) carrying one loss-of-function allele (*1/*2, *1/*4); (3) poor metabolizers (PMs) carrying two loss-of-function alleles (*2/*2, *2/*4, *4/*4); (4) rapid metabolizers (RMs) for alleles (*1/*17); and (5) ultrarapid metabolizers (UMs) for alleles (*17/*17) [10,11,12,13].

Compared to other antidepressant agents, SSRIs were initially considered to be almost free of side effects [14]. However, post-marketing data and broad-based experience with SSRIs have captured more frequent and new types of side effects that were not reported in the original clinical trials [15,16]. Among others, the trial designs, definitions and reporting of adverse events, and short durations of the studies probably caused underestimations of the real numbers of side effects.

The current study was designed to explore possible correlations between the CYP2C19 genotype-based metabolizing groups and the real-life, reported, long-term, adverse effects and efficacies in patients prescribed Citalopram and Escitalopram therapy. Innovatively, we tested the assumption that genetic variation can be used to identify homogeneous subgroups within the larger population applying the latent-class model. 

## 2. Methods

### 2.1. Study Design and Setting

We conducted a retrospective, longitudinal cohort study. The study used the electronic health record (EHR) database of Leumit Health Care Services (LHS). LHS is a health maintenance organization from Israel. LHS’s database includes demographics of the members and their complete medical records, including visits to physicians and laboratory tests, emergency room referrals and hospitalizations, chronic medications and dosing, and documented side effects. The diagnostic codes used in the medical records follow the definition of the International Classification of Diseases, Ninth Revision, Clinical Modification (ICD-9-CM). This study was approved by the Asaf-Harofeh Medical Center and the LHS institutional review committee (protocol number 0225-18-ASF). Due to the retrospective nature of this study, the need for informed consent was waived.

The LHS computerized database was reviewed retrospectively for the period from 1 January 2013 to 31 December 2018 for patients diagnosed with MDD by 1 January 2013, prescribed SSRIs at an adequate dose for ≥ 4 weeks, and with available blood-derived pharmacogenetic profiling for CYP2C19 isoenzymes (already tested in a former study from LHS). Patients’ characteristics, medical histories, and diagnostic workup and medical treatment details were retrieved. A detailed analysis of their medication usage was also performed. The follow-up period lasted five years, from 1 January 2013 to 31 December 2018, and it was divided into 24 quarters.

### 2.2. Eligible Patients

All patients aged > 18 years with a documented ICD-9-MC MDD diagnosis (codes 296.21–296.36 and 296.3) established twice in 2013 by a board-certified psychiatrist and who had available blood test results for CYP2C19 genotyping were included in the study. The SSRI agent had to be documented as having been acquired for the whole trial period. It should be noted that none of the patients was treated with MAOIs. Exclusion criteria included pregnancy or lactation, malignancy, recent (<6 months) or current history of substance use disorder, noncompliance with treatment, and lifetime history of psychosis, hepatitis A/B/C infection, or HIV. Patients with depression secondary to other diseases, such as Parkinson’s disease, stroke, dementia, or active and chronic infectious diseases, were also excluded from this study.

Comorbidities were identified via ICD-9-CM codes documented by the attending board-certified physician. A body mass index (BMI) > 30 kg/m^2^ was considered obese. All patients were fully medically followed up by the attending physician and prescribed Citalopram or Escitalopram monotherapy, and no patient was lost for follow-up. Each included patient reached the study endpoint only if they were transferred to other health care services, died, or remained at the end of the study data collection period.

### 2.3. Classification of Side Effects and Main Outcomes

Citalopram- or Escitalopram-related side effects were classified as serious or non-serious side effects. A serious side effect in human drug trials is defined as any untoward medical occurrence that, at any dose, results in death, hospitalization, life-threatening events, or referral to a specialist physician, or that causes the prolongation of existing hospitalization.

### 2.4. Data Collection

We collected the social and demographic information from the electronic medical records. The electronic medical records of Leumit include data from multiple sources: records of primary care physicians, community specialty clinics, hospitalizations, laboratories, and pharmacies. A registry of chronic disease diagnoses was compiled from these data sources. Diagnoses were captured in the registry via diagnosis-specific algorithms, employing the International Classification of Diseases, Ninth Revision (ICD-9), code reading, text reading, laboratory test results, and disease-specific drug usage. A record of the data sources and dates used to establish the diagnosis from any source, with the earliest recorded date, was considered to be the defining date of diagnosis. The primary measure of medication adherence, the PDC (proportion of days covered), was calculated by extracting retail pharmacy data. In addition, for each patient, we calculated the defined daily dose (DDD): the assumed average maintenance dose per day for a drug used for its main indication in adults.

Patients’ identifications and blood-derived pharmacogenetic profiles were previously coded to identification codes and tested at the Genelex Laboratory in the United States. 

We followed the patients until the occurrence of any one of the following: severe adverse effects; death; the end of the study period (31 December 2018); transfer to other health care services; switching to another antidepressant; the discontinuation of treatment with the drug. 

### 2.5. Statistical Analysis

A proportional-odds model with repeated measurements was constructed to estimate the effect of the CYP2C19 genotype on the risk for adverse events among Citalopram and Escitalopram users. The dependent variable was an ordered variable consisting of three levels: “no side effects”, “mild side effects”, and “severe side effects”. The independent or explanatory variables were pre-specified based on a literature survey. The pre-specified explanatory variables included age, medication, daily defined dose, proportion of days covered, a socio-economic score, and the CYP2C19 genotype modeled as an ordinal variable with four levels. A multilevel-modeling approach was used with two levels with varying intercepts: level 2, the individual patient, and level 1, the follow-up appointment. 

The model was fitted using the “brms” package in R (version 0.10.0) [17,18]. The default priors in the “brms” package (version 0.10.0) were used. Briefly, the “brms” default sets improper flat priors over the reals on the population-level effects, a half Student-t prior with 3 degrees of freedom, and a scale parameter that depends on the standard deviation of the response after applying the link function to the group-level effects. The “brms” package (version 0.10.0) utilizes the No-U-Turn Sampler (NUTS) to approximate the posterior distribution of the coefficients of the explanatory variables. The sampling was performed with four chains, with 2000 iterations in each chain (warmup: 1000 iterations) and an adapt delta value of 0.95. The coefficient estimates of the explanatory variables and the 95% posterior distribution upper and lower limits (i.e., credible intervals) for each coefficient were reported. 

To identify groups of clinical interest, we used the following four indicator variables: the presence of side effects, good adherence (defined as PDC > 0.8), medication (either Citalopram or Escitalopram), and any event of a change in medication or dose modification. We used latent-class analysis (LCA). The number of classes was restricted to two due to the limited sample size. Moreover, the CYP2C19 genotype as a predictor of class membership was simultaneously estimated. The CYP2C19 genotype coefficient estimate and 95% confidence interval were calculated. LCA analysis was performed using the “poLCA” package in R (version 1.3) [19].

## 3. Results

### 3.1. Patient Characteristics

A total of 283 eligible patients were allocated. Table 1 summarizes the characteristics of the patients, including the demographics, laboratory tests, and comorbidities, all stratified by CYP2C19 category. The patients’ ages ranged from 23 years to 93 years, with a median of 66 years. All the study patients had a diagnosis of MDD before the Citalopram or Escitalopram initiation date. A total of 196 (69%) patients were women, 255 (89.8%) were Jewish, 207 (72.8%) were using Escitalopram, and 77 (27.2%) were using Citalopram. The range of the follow-up was between 0.4 and 6 years, with a median follow-up period of 5.1 years (IQR: 4.6–5.9). Among the study cohort, 33% reported smoking cigarettes and non-reported the use of concomitant herbal medicines or any known potent CYP2C19 inducers or inhibitors. The distribution of the CYP2C19 genotyping among the participants is included in Table 2. 

### 3.2. Distribution and Nature of Drug Events

A total of 1032 side effects were extracted during the follow-up period. Of these, 261 (25.3%) were gastrointestinal-related; 455 (44.1%) were neurological; 89 (8.6%) were classified as metabolic and endocrine; 45 (4.4%) were in the genitourinary system; 58 (5.6%) were neuromuscular and skeletal; 58 (5.6%) were considered cardiovascular; 47 (4.6%) were ophthalmic; and 19 (1.8%) were dermatological. Table 3 summarizes the list and frequencies of the reported side effects, including the median time elapsed from drug initiation to side effect documentation. According to the inferred phenotype, we found that UM patients experienced fewer side effects compared to patients who were classified as NMs or IMs; the higher the CYP2C19 metabolizer status, the fewer average side effects encountered per patient. The distribution of the different side effects in relation to the patients’ CYP2C19 phenotypes is presented in Table 4.

### 3.3. Associations between Side Effects and Covariates

The phenotype was found to be significantly associated with side effects, adjusting for the four independent variables involved in the multivariable, longitudinal, proportional-odds model. For every CYP2C19 activity score unit, the odds for side effects decreased by a factor of 0.69 (OR = 0.69; 95% credible interval: 0.51–0.84). The odds of experiencing non-severe side effects decreased by 0.73 with each CYP2C19 activity score unit increase, and, assuming an uninformative prior, there was a 95% probability that the actual effect was in the interval of 0.56–0.89. For patients with a prescribed daily dose/defined daily dose (DDD) above one, the odds for side effects were 2.86 (95% credible interval: 1.82–4.46). There was no statistically significant difference in the risk of developing side effects depending on the type of drug (Escitalopram/Citalopram). 

### 3.4. CYP 2C19 Phenotype as Predictor of Latent-Class Membership

**Figure 1:**
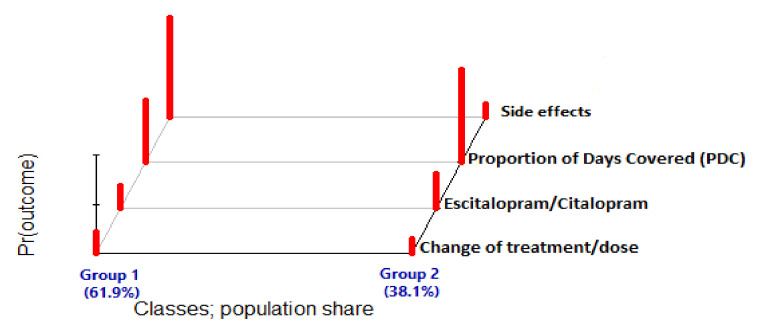
Latent-class membership. Subgroup 1 of the latent class is characterized by many side effects, low adherence, and frequent treatment changes. Subgroup 2 of the latent class is characterized by few side effects, high adherence, and few treatment changes.

The study population was divided into two homogeneous groups according to four categorical indicator variables: the presence of side effects, a good adherence level (defined as a proportion of days covered > 0.8), type of medication (either Citalopram or Escitalopram), and any issues of changes in the existing prescription (type of medication or dosage modification) (Figure 1). The first group, which comprised 61.8% of the study cohort, was characterized by frequent side effects (100%), low adherence levels (62.8%), and substantial changes in the existing agents (21.9%). The second group contained 38.2% of the study cohort and was characterized by low rates of side effects (12.3%), good adherence levels (93.8%), and few changes in the existing agents (14.2%). Notably, the prescribed medications (Citalopram or Escitalopram) were similar in both groups.

**Figure 2:**
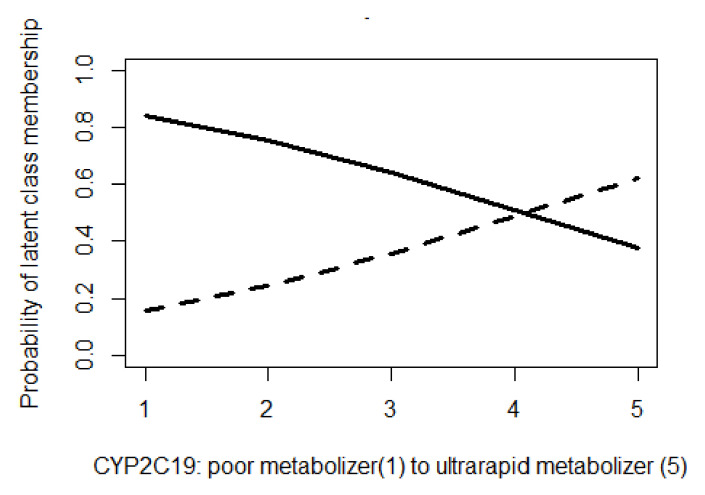
CYP2C19 phenotype as predictor of latent-class membership. Solid line represents probability of latent-class “high side effects, low adherence, high treatment change” membership as function of phenotype. Dotted line represents probability of latent-class “low side effects, high adherence, low treatment change” membership as function of phenotype.

The group membership as a function of the CYP2C19 phenotype is presented in Figure 2; the higher the CYP2C19 metabolic range, the higher the probability for second-group membership, and vice versa.

## 4. Discussion

This study found a robust association between the genotype-based metabolizing group of CYP2C19 and the risk of experiencing adverse drug effects in patients treated with Citalopram or Escitalopram. To the best of our knowledge, this is the first long-term, real-life study that has investigated the impact of the genetic variability in CYP2C19 activity on the tolerability and efficacy profiles of patients prescribed Citalopram or Escitalopram, applying latent-group analysis to uncover latent practical subgroups. We found that the probability of experiencing side effects decreased with each one-degree increase in metabolic activity, and, when the DDD increased, the probability of side effects increased. In addition, the research group was divided into two groups with similar overall characteristics. The CYP2C19-related metabolizing group proved to be an important predictor of group membership. The low metabolizers had low adherence, frequent changes in treatment regimens, and more side effects than the rapid metabolizers; in other words, in the real-world model, the slow metabolizers predicted the “expected” low tolerability, but this was also accompanied by “surprisingly” low-efficacy features compared to the fast metabolizers. Successive studies have supported the association between CYP2C19 polymorphism and the risk of adverse effects under treatment with antidepressants, while others have not. In a non-interventional study design, Jucik et al. [20] showed that elevated CYP2C19 expression was associated with depressive symptoms and higher tendencies towards suicidal impulses in humans. In a retrospective study using data from a database that included 2087 CYP2C19-genotyped patients under Escitalopram treatment, patients were divided into subgroups based on CYP2C19 genotype [21]. It was found that the higher the CYP2C19 metabolizer state, the lower the Escitalopram serum concentration. Moreover, therapeutic failure, measured by the switching of antidepressant therapy, was the highest among the poor and ultrarapid metabolizers. The authors attributed this observation to the presumptive high occurrences of side effects and subtherapeutic Escitalopram concentrations in the poor and ultrarapid metabolizers, respectively. Several constraints limit the reliability of the results:

- The unavailability of the patients’ volumes of distribution, which inversely affect the drug concentration;
- No information on comedications with potential interactions with Escitalopram;
- The absence of exact information on the diagnosis and treatment outcomes, including the patients’ side effects, adherence, comorbidities, renal/liver function, Escitalopram continuation, and timing of drug switching.

The above constraints potentially limit the validity of the findings and reinforce the superiority of our controlled-setting study design. 

In another large meta-analysis, totals of 2558 patients and 2037 patients were analyzed for efficacies and side effects of Citalopram/Escitalopram, respectively. Under similar antidepressant doses, the poor metabolizers (PMs) exhibited greater symptom improvements and higher remission rates compared to the extensive metabolizers (EMs). At weeks 2–4, the PMs showed higher occurrences of side effects. However, no differences were disclosed at week 9 or in the total side effect burden. The documented side effect profiles did not translate to a higher risk of dropout at week 4 in the PMs compared to the EMs. Overall, the message of this short follow-up study might be misleading, pointing towards the superiority of PMs, as the side effect burden “dissipates” at 9 weeks, proving superior efficacy relative to EMs [22].

In another study, the geometric mean serum concentration of Escitalopram was 42% lower in patients homozygous for CYP2C19*17 (*p* < 0.01) and 5.7-fold higher in subjects homozygous for defective CYP2C19 alleles (*p* < 0.001) [23]. The results support that a homozygous CYP2C19*17 genotype is associated with a lower serum concentration of Escitalopram, which might imply an increased risk of therapeutic failure despite the smaller risk of side effects. 

Campos et al. [24] retrospectively tested 9500 MDD patients for the impact of the CYP2C19 metabolizer status on the SSRI response, using their perceived effectiveness as the binary response. Despite the overall results highlighting correlations between the metabolizer status and both the tolerability and safety, the results were not significantly powered, mainly due to the study design and the absence of longitudinal follow-up. Similar results were reported by Calabro et al. [25], who studied 1239 patients with MDD for a correlation between the CYP2C19 metabolizing status and antidepressant safety profile. They found a positive correlation between PMs and drug efficacy, while presenting higher numbers of side effects. These studies have several limitations: no corrections were performed for the smoking status as a confounder, and the study designs were cross-sectional and thus lacked the capacity to calculate the dropout rate due to side effects in longitudinal follow-up designs. Overall, the above studies have a major drawback: they do not represent the real-world setting mainly due to their cross-sectional designs and short follow-up durations, limiting their ability to identify and quantify the side effects (especially rare side effects), adherence to treatment, and long-term treatment dropout or success. 

One of the strengths of our study is its real-world, long-term patient management, with the study population representing a wide range of the Israeli population, including Jewish and Arab patients. Under this controlled setting, we had no missing data throughout the follow-up period.

The current study recapitulates real-world data by capturing the longitudinal long-term experience as reported and handled by the treating physician. Our findings are consistent with other studies that have reported an increased risk of side effects among poor metabolizers compared to rapid or ultrarapid metabolizers (Table 4). Previous cross-sectional/short-term studies have demonstrated a substantial trend toward lower efficacies with higher metabolizer phenotypes, ascribed to rapid drug elimination. Nevertheless, our real-time data applying the latent-class membership model showed less overall drug class switching and higher adherence with the higher metabolizer phenotypes. The multilevel, proportional-odds model allowed for an analysis that used all the available data. This fact probably reflects the lower rate of non-responders among higher metabolizers.

This study has some limitations. First, despite the multivariate model adjustment that accounted for relevant potential confounders, some residual confounding might exist due to the study’s retrospective nature. For example, the severity of the depression and environmental factors (e.g., chronic stress) may have affected the likelihood of experiencing side effects beyond the influence of the genetic background. The second limitation is that it was a small population sample, limiting the generalizability of the findings. Finally, a causal relationship between the CYP2C19 phenotype and the occurrence of side effects cannot be generated due to the study’s observational design.

## 5. Conclusions

The CYP2C19 metabolizer status helped to explain and predict the likelihood of experiencing side effects in Israeli patients with depressive disorders who were prescribed Es/Citalopram. The phenotype of CYP2C19 was an important predictor of the group membership. Patients with low adherence, frequent changes in treatment regimens, and more side effects were more likely to be slow metabolizers. The results support the potential clinical utility of CYP2C19 genotyping in Escitalopram and Citalopram therapy personalization. Our findings suggest that dosing Es/Citalopram based on the CYP2C19 metabolizer status could potentially improve the safety and efficacy while treating depressive patients. Future clinical studies with pharmacokinetic measurements should follow our results.

## Figures and Tables

**Table 1:** Demographic and clinical characteristics stratified by phenotype.

Poor Metabolizers (*n* = 5)	Intermediate Metabolizers (*n* = 63)	Normal Metabolizers (*n* = 127)	Rapid Metabolizers (*n* = 76)	Ultrarapid Metabolizers (*n* = 13)	Total (*n* = 284)	Reported Result	Patient Information and History
66 (15.9)	65 (14.1)	65 (11.8)	66 (12.4)	64 (16.3)	66 (12.7)	Mean (SD)	Age
30 (5.7)	29.6 (5.5)	30.1 (5.5)	30.6 (5.9)	29.2 (5.05)	30 (5.62)	Mean (SD)	Body mass index
3 (60)	19 (30.1)	38 (30)	25 (32.9)	3 (23)	88 (31)	Males (n (%))	Gender
2 (40)	44 (69.9)	89 (70)	51 (67.1)	10 (77)	196 (69)	Females (n (%))	
5 (100)	59 (93.6)	114 (89.7)	66 (86.8)	11 (84.6)	255 (89.8)	Jewish (n (%))	Race ^¶^
0 (0)	4 (6.4)	13 (10.2)	10 (13.2)	2 (15.4)	29 (10.2)	Arabs (n (%))	
4 (80)	59 (93.6)	124 (97.6)	74 (97.4)	13 (100)	274 (96.5)	City (n (%))	Settlement
1 (20)	4 (6.4)	3 (2.4)	2 (2.6)	-	10 (3.5)	Village (n (%))	
4 (2–9)	6 (3–8)	6 (0–10)	5 (3–9)	5 (4–7)	6 (0–10)	Median (CI)	Socio Economic Index
2 (40)	17 (27)	45 (35.4)	24 (31.6)	3 (23)	91 (32%)	n (%)	Smoking
4 (80)	45 (71.4)	94 (74)	54 (71)	10 (77)	207 (72.8)	Escitalopram (n (%))	Drugs
1 (20)	18 (28.6)	33 (26)	22 (29)	3 (23)	77 (27.2)	Citalopram (n (%))	
-	28 (44.4)	45 (35.4)	29 (38.1)	3 (23)	105 (37)	n (%)	Diabetes
-	22 (35)	39 (30.7)	32 (42.1)	4 (30.7)	97 (34.1)	n (%)	Hypertension
-	4 (6.4)	-	9 (11.8)	1 (7.7)	22 (7.7)	n (%)	Congestive heart failure
-	12 (19)	11 (8.7)	24 (31.6)	-	76 (26.8)	n (%)	Hyperlipidemia
-	16 (25.4)	16 (12.6)	18 (23.7)	2 (15.4)	52 (18.3)	n (%)	Coronary artery disease
-	5 (8)	9 (7.1)	9 (11.8)	2 (15.4)	25 (8.8)	n (%)	Asthma/COPD ^Ϯ^
-	-	1 (0.8)	4 (5.3)	1 (7.7)	6 (2.1)	n (%)	Epilepsy/seizures
2 (40)	8 (12.7)	13 (10.2)	3 (3.9)	3 (23)	29 (10.2)	n (%)	Hypothyroidism
-	1 (1.6)	10 (7.9)	6 (7.9)	-	17 (6)	n (%)	CVA/TIA ^Ϯ^
1 (20)	7 (11.1)	10 (7.9)	5 (6.6)	4 (30.7)	27 (9.5)	n (%)	Osteoporosis
-	10 (15.9)	8 (6.3)	12 (15.8)	2 (15.4)	32 (11.3)	n (%)	GERD ^Ϯ^
1 (20)	5 (8)	8 (6.3)	-	1 (7.7)	25 (8.8)	n (%)	Chronic kidney disease
2 (40)	36 (57.1)	68 (53.5)	48 (63.2)	8 (61.5)	156	>80 mL/min	Creatinine clearance—mL/min ^ⱡ^
2 (40)	23 (36.5)	50 (39.4)	19 (25)	4 (30.7)	98	50–80 mL/min	
1 (20)	4 (6.4)	7 (5.5)	8 (10.5)	1 (7.7)	27	30–49 mL/min	
-	-	2 (1.6)	1 (1.3)	-	3	<30 mL/min	
2 (40)	3 (4.8)	13 (10.2)	8 (10.5)	4 (30.7)	30 (10.6)	n (%)	Atrial fibrillation
-	10 (15.9)	15 (11.8)	10 (13.2)	3 (23)	38 (13.4)	n (%)	Anxiety
-	-	7 (5.5)	-	-	7 (2.5)	n (%)	Schizophrenia
22.2 (± 2.5)	22.6 (± 0.9)	22.5 (± 0.53)	22.2 (± 0.76)	20.1 (± 1.2)	22.3 (± 0.37)	Mean (± SE)	AST ^Ϯ^
23 (± 2.5)	19 (± 1.0)	20 (± 0.8)	19 (± 1.04)	15 (± 1.3)	19 (± 0.52)	Mean (± SE)	ALT ^Ϯ^
21 (± 2.1)	28 (± 2.7)	34.4 (± 2.4)	36.1 (± 5.2)	23.7 (± 3.1)	32.7 (± 1.9)	Mean (± SE)	GGT ^Ϯ^
1.17 (± 0.3)	0.8 (± 0.04)	0.89 (± 0.05)	0.92 (± 0.06)	0.81 (± 0.07)	0.89 (± 0.03)	Mean (± SE)	Creatinine

^¶^ Race was reported by the patient. ^Ϯ^ COPD—chronic obstructive pulmonary disease; CVA—cerebral vascular accident; TIA—transient ischemic attack; GERD—gastroesophageal reflux disease; AST—aspartate transaminase; ALT—alanine transaminase; GGT—gamma-glutamyl transferase; ⱡ: the creatinine clearance was calculated with the Cockcroft–Gault formula.

**Table 2:** CYP2C19 genotype and phenotype frequencies in patients.

Phenotype	Genotype	Patients (n (%))
Extensive/normal metabolizers	CYP2C19*1/*1	127 (44.7)
Intermediate metabolizers	CYP2C19*1/*2	46 (16.2)
	CYP2C19*2/*17	17 (5.9)
Poor metabolizers	CYP2C19*2/*2	5 (1.8)
Rapid metabolizers	CYP2C19*1/*17	76 (26.7)
Ultrarapid metabolizers	CYP2C19*17/*17	13 (4.6)

**Table 3:** Occurrence of adverse drug reactions classified by body systems, including median time elapsed from drug initiation to side effect occurrence.

System Adverse Reaction		Total Cohort (n = 284) (*n* (%))	Escitalopram (*n* = 207) (*n* (%))	Citalopram (*n* = 77) (*n* (%))	Median Time to Event (Weeks)
Gastrointestinal (*n* = 261)	Abdominal pain	127 (48.7)	94 (36.0)	33 (12.7)	4 (2–7)
	Constipation	60 (23.0)	56 (21.4)	4 (1.5)	
	Diarrhea	44 (16.8)	34 (13.0)	10 (3.8)	
	Nausea and vomiting	30 (11.5)	26 (10.0)	4 (1.5)	
Nervous system (*n* = 455)	Dizziness	53 (11.6)	37 (8.1)	16 (3.5)	13 (9–18)
	Headache	65 (14.3)	55 (12.1)	10 (2.2)	
	Insomnia	96 (21.1)	77 (16.9)	19 (4.2)	
	Anxiety	105 (23.1)	75 (16.5)	30 (6.6)	
	Agitation	17 (3.7)	16 (3.5)	1 (0.2)	
	Vertigo	22 (4.8)	15 (3.3)	7 (1.5)	
	Fatigue	37 (8.1)	21 (4.6)	16 (3.5)	
	Drowsiness	43 (9.4)	28 (6.1)	15 (3.3)	
	Depressive episode	17 (3.7)	9 (2.0)	8 (1.7)	
Endocrine and metabolic (*n* = 89)	Weight loss	17 (19.1)	12 (13.5)	5 (5.6)	27 (17–39)
	Loss of appetite	2 (2.2)	1 (1.1)	1 (1.1)	
	Sexual dysfunction	8 (9.0)	5 (5.6)	3 (3.4)	
	Weight gain	47 (52.8)	32 (36.0)	15 (16.8)	
	Hypoglycemia	6 (6.7)	4 (4.5)	2 (2.2)	
	Hyponatremia and SIADH	9 (10.1)	6 (6.7)	3 (3.4)	
Genitourinary (*n* = 45)	Urinary frequency	28 (62.2)	25 (55.5)	3 (6.7)	21 (14–29)
	Urinary retention	17 (37.7)	2 (4.4)	15 (33.3)	
Neuromuscular and skeletal (*n* = 58)	Parkinsonism	10 (17.2)	9 (15.5)	1 (1.7)	38 (29–49)
	Tardive dyskinesia	2 (3.4)	1 (1.7)	1 (1.7)	
	Muscle weakness	2 (3.4)	1 (1.7)	1 (1.7)	
	Tremor	18 (31.0)	13 (22.4)	5 (8.6)	
	Myalgia and myositis	26 (44.8)	16 (27.6)	10 (17.2)	
Cardiovascular system (*n* = 58)	Orthostatic hypotension	8 (13.8)	5 (8.6)	3 (5.2)	28 (21–36)
	Palpitation and tachycardia	27 (46.5)	16 (27.6)	11 (18.9)	
	Syncope	18 (31.0)	12 (20.7)	6 (10.3)	
	Prolonged QT	5 (8.6)	3 (5.2)	2 (3.4)	
Ophthalmic (*n* = 47)	Visual disturbance	30 (63.8)	19 (9.2)	11 (14.3)	47 (36–59)
	Blurred vision	17 (36.1)	10 (17.2)	7 (14.9)	
Dermatologic (*n* = 19)	Skin rash	19 (100)	13 (68.4)	6 (31.6)	52 (38–64)

**Table 4:** Adverse reactions stratified by CYP2C19 metabolizer status. SE—side effects.

Escitalopram/Citalopram	Poor Metabolizers (*n* = 5)	Intermediate Metabolizers (*n* = 63)	Normal Metabolizers (*n* = 127)	Rapid Metabolizers (*n* = 76)	Ultrarapid Metabolizers (*n* = 13)
Gastrointestinal	8	90	107	45	11
Nervous system	26	114	232	75	8
Endocrine and metabolic	6	39	25	14	5
Genitourinary	4	21	12	8	0
Neuromuscular and skeletal	1	15	29	12	1
Cardiovascular system	3	13	35	6	1
Ophthalmic	1	19	22	5	0
Dermatologic	4	5	8	2	0
Average SE per patient	10.6	5	3.7	2.2	2

## Data Availability

All data presented in this manuscript are available from the corresponding author upon reasonable request.
